# Measuring effectiveness of the cervical cancer vaccine in an Australian setting (the VACCINE study)

**DOI:** 10.1186/1471-2407-13-296

**Published:** 2013-06-19

**Authors:** Elisa J Young, Sepehr N Tabrizi, Julia ML Brotherton, John D Wark, Jan Pyman, Marion Saville, C David Wrede, Yasmin Jayasinghe, Jeffrey Tan, Dorota M Gertig, Marian Pitts, Suzanne M Garland

**Affiliations:** 1 Department of Microbiology and Infectious Diseases, Royal Women’s Hospital, Level 1, Building 404, Bio 21 Institute, 30 Flemington Road, Parkville, Melbourne, VIC 3052, Australia; 2 VCS Incorporated, 265 Faraday St, Carlton, Melbourne, VIC 3053, Australia; 3 La Trobe University, Australian Research Centre for Sex, Health & Society, Level 1, 215 Franklin Street, Melbourne, VIC 3000, Australia; 4 Dept of Oncology/Dysplasia RWH, 20 Flemington Road, Parkville, Melbourne, VIC 3052, Australia; 5 Dept of Medicine, Bone and Mineral Medicine, The University of Melbourne and The Royal Melbourne Hospital, Level 4, Clinical Sciences Building, Royal Pde, Parkville, Melbourne, VIC 3050, Australia; 6 Anatomical Pathology, The Royal Women’s Hospital, 20 Flemington Road Parkville, Vic 3052, Australia; 7 Department of Microbiology and Infectious Diseases, Murdoch Childrens Research Institute, Anatomical Pathology, The Royal Women’s Hospital., Level 1, Building 404, Bio 21 Institute, 20 Flemington Road Parkville, Melbourne, VIC 3052, Australia; 8 Department of Obstetrics and Gynaecology University of Melbourne; 9 School of Population and Global Health, University of Melbourne, Melbourne, Australia

**Keywords:** Human papillomavirus, Cervical cancer, CIN3, LCM, Pap smears

## Abstract

**Background:**

The quadrivalent human papillomavirus vaccine has been provided in Australia through the National Human Papillomavirus Vaccination Program since April 2007. National registry data demonstrates good coverage of the vaccine, with 73% of school-aged girls having received all three doses. To evaluate the effectiveness of the program, we propose a two-pronged approach. In one (sub study A), the prevalence of the vaccine-targeted human papillomavirus genotypes in a population cohort is being estimated, and will be analysed in relation to vaccination status, cervical cytology screening status, demographic, social, behavioural, medical and clinical factors. In sub study B, the distribution of human papillomavirus genotypes detected in high grade cervical intraepithelial neoplastic lesions from vaccine eligible women is being assessed.

**Methods/Design:**

Sub Study A involves the recruitment of 1569 women aged 18–25, residing in Victoria, Australia, through Facebook advertising. Women who are sexually active are being asked to provide a self-collected vaginal swab, collected at home and posted into the study centre, where human papillomavirus DNA detection and genotyping is performed. Participants also complete an online questionnaire regarding sexual history, experience with, knowledge of, and attitudes towards human papillomavirus, the human papillomavirus vaccine, and cervical screening.

Sub Study B will involve the collection of 500 cervical biopsies, positively identified as containing high grade cervical intraepithelial neoplastic lesions and/or adenocarcinoma in situ. Five serial sections are being taken from each case: sections 1 and 5 are being assessed to confirm the presence of the high grade cervical intraepithelial neoplastic lesions or adenocarcinoma in situ; human papillomavirus genotyping is performed on sections 2 and 3; single lesions are excised from section 4 using laser capture microdissection to specifically define causality of a human papillomavirus genotyping of each specific lesion.

**Discussion:**

Australia is well placed to gain a clear and early insight into the effectiveness of the human papillomavirus vaccine in reducing the prevalence of human papillomavirus infection in young women, and any subsequent reduction in the prevalence of pre-cancerous cervical lesions, specifically high grade cervical intraepithelial neoplasia lesions, particularly of vaccine related types. The findings of a successful population based human papillomavirus program will have wide-reaching translational benefits across the globe.

## Background

Cervical cancer is the third most common cancer among women worldwide [1]. Almost half a million newly diagnosed cases and close to 275,000 deaths occur annually [1]. However, in Australia, due to its successful comprehensive National Cervical Screening Program, it ranks in 13^th^ place among the most common cancers in women [2]. Nevertheless, morbidity associated with persistent high-risk human papillomavirus (HPV) infection, primarily the detection and treatment of precancerous abnormalities, still poses a significant physical, emotional, and financial burden [3-5].

In April 2007, Australia became the first country to commence a government funded National HPV Vaccination Program, providing three doses of the quadrivalent HPV vaccine Gardasil^TM^, for all females aged between 12 and 26 years of age. This was largely a school-based vaccination program (age 12–18 years), with vaccination of young women up to 26 years, delivered through schools, general practices and community-based programs. With the catch-up program finishing in December 2009, girls aged 12–13 have continued to be offered HPV vaccination through the National Immunisation Program, and from 2013 boys too are being offered vaccine through the government funded school-based program.

The prophylactic quadrivalent HPV vaccine, was shown in phase 3 trials to be almost 100% effective for vaccine-related HPV types in preventing their respective related disease, including genital warts, cervical intraepithelial neoplasia (CIN), vulvar intraepithelial neoplasia (VIN), vaginal intraepithelial neoplasia (VAIN) in young women naïve to these HPV types targeted by the vaccine [6-8]. Hence it is predicted that where vaccines are adopted in public health programs with good coverage that prevalence of HPV types 6, 11, 16 and 18 will fall [9], as well as the incidence of these vaccine HPV type related diseases. Collectively, HPV-16 and HPV-18 are particularly virulent and are responsible for approximately 70% of all cervical cancer cases worldwide [10] and closer to 80% in North America and Oceania [11,12]. Moreover these two types are responsible for ~50% of high grade CIN lesions globally [13,14].

In Australia, where coverage in school-age girls for 3 doses is reported at 73% [15], detection of vaccine-targeted HPV genotypes at the cervix has significantly fallen in young women presenting to family clinics for Pap tests, reducing from 28.7% in the pre-vaccine sample to 6.7% in the post-vaccine sample. Furthermore, in the interim analysis of this post vaccine study this effect was shown to be greatest in women who had received at least one dose of the vaccine [9]. Similarly, in Victoria, Australia a significant reduction in the incidence of histologically diagnosed high-grade cervical dysplasia lesions in those of vaccine-eligible age has been reported [16]; nationally too there has been a significant fall in high-grade abnormalities in those aged under 20 years [2]. Rates of genital warts, which are largely caused by HPV-6 and HPV-11, have fallen by 90% in young women of vaccine-eligible age presenting to sexual health clinics [17]. The decline in genital warts was seen as early as 2009, two years after the introduction of the vaccine, with one study showing a 59% decline in the monthly presentation for warts among women <28 years but not among women >28 years [18,19]. These data suggest that HPV vaccination is rapidly reducing incidence of infection with targeted-HPV types.

The long-term benefit of the vaccine in preventing HPV-16/18 related cervical cancer will not be seen for decades given the long incubation from CIN3 to cancer [20]. Whilst Australia has an 8-point plan for monitoring the vaccine program and control of HPV and related diseases [20], the Vaccine Against Cervical Cancer Impact and Effectiveness (VACCINE) study is focussing on two components of this. One is to measure circulating genotypes among young women in cohorts who were offered HPV vaccination (who are at a time of peak risk for the acquisition of new HPV infection) and compare these data with the available pre-vaccination data. This will provide an early indicator of likely HPV vaccine effectiveness [21]. Secondly, as diagnosis of screen detected CIN2/CIN3 peaks in the 20–29 year old age group in Australia, and infection occurs very shortly after sexual debut, we are prospectively typing HPV in CIN3 lesions diagnosed in women within this age group to ascertain the current causative HPV types in the post vaccine implementation period.

A World Health Organization (WHO) working group [22] considered high grade cervical lesions (CIN2+/3+/adenocarcinoma in situ (ACIS)) appropriate alternative endpoints for cervical cancer in HPV vaccination studies for the following reasons: high grade cervical lesions are obligate precursors of cervical cancer; high grade cervical lesions are closely associated in temporal sequence to the development of invasive cervical cancer; high grade cervical lesions are associated with a high risk of development of invasive cervical cancer; and reductions in incidence or treatment of high grade cervical lesions are shown to result in a reduction in risk of invasive cervical cancer.

### Study objectives

This paper describes a two-pronged approach to assessing the effectiveness of the Australian HPV vaccination program in reducing both the prevalence of vaccine-targeted HPV genotypes in a population cohort (sub study A); and assessing the proportion of CIN3 cases positive for vaccine-targeted HPV genotypes in a biopsy cohort (sub study B).

In sub-study A the objective is determining the prevalence of vaccine-targeted HPV carriage [6, 11, 16 and/or 18] in young women aged 18 to 25 years living in Victoria, Australia. In addition, vaccination status along with demographic, social, behavioural, medical and clinical factors, including HPV related knowledge, will be assessed to identify predictors of genital HPV DNA in the cohort, HPV vaccination (considering single dose through to full vaccination, both self-reported and as held on the National HPV Register), and participation in cervical cytology screening (Pap testing).

In sub-study B, the objective is to assess HPV genotypes in prospectively and systematically collected CIN3 biopsies, in combination with vaccination status where available, to assess the distribution of HPV genotypes detected in CIN3 biopsies from HPV vaccine eligible women (born after 31^st^ June 1981) in Victoria. CIN3 has now been demonstrated to be the true precursor lesion to cancer, unlike CIN2 which is a poorly reproducible category of lesions which contains a mixture of acute HPV infection and true CIN3 [23].

For both sub-studies, potential vaccine type cross-protection and genotype replacement will also be considered. Possible indication of vaccine cross-protection will be measured through assessing the prevalence of HPV-16 and 18 related, but non-vaccine targeted HPV types, e.g. HPV-31 and HPV-45 respectively; and any early indicators of possible genotype replacement will be measured through assessment of the post vaccine prevalence of all other high risk HPVs in the context of the absolute rate of CIN3 lesions detected over time in Victoria.

## Methods

The study protocol was approved by the Royal Women’s Hospital Human Research Ethics Committee, and is being carried out according to the National Statement on Ethical Conduct in Research Involving Humans (June 1999) produced by the National Health and Medical Research Council of Australia.

### Methods: sub study A

#### Recruitment

A sample of women aged 18–25 years are being recruited via a Facebook advertising campaign. This recruitment and data collection strategy was piloted, and established to achieve a broadly demographically-representative sample in this age bracket, by comparison with census data obtained from the Australian Bureau of Statistics [24]. Participants are asked to complete a questionnaire about their sexual history and their experience with, knowledge of, and attitudes towards HPV, the HPV vaccine, and cervical screening. Participants are requested to provide information regarding their HPV vaccination status, as well as provide consent for us to obtain their HPV vaccination history from the National HPV Vaccination Program Register (NHVPR). Women who are sexually active are asked to provide a self-collected vaginal swab using a swab kit sent in the post. The use of self-collected sampling for HPV DNA testing has been shown to compare favourably with physician-collected samples and cytology, with concordances between 92 and 96% demonstrated [25-28].

### HPV DNA genotyping

HPV detection and genotyping is being performed at the Western Pacific Regional HPV Labnet Laboratory located at the Royal Women’s Hospital, Melbourne, Australia. Each self-collected Flocked swab is rotated in 400μL of phosphate buffered saline (PBS) and 200μL utilized for DNA extraction using the automated MagNA Pure LC isolation and purification system (Roche Molecular Systems, Pleasanton, CA) with the DNA-I isolation kit. Following nucleic acid isolation, all samples are being tested for the presence of mucosal HPV DNA using L1 consensus primer set PGMY09-PGMY11 [29]. PCR products are then detected by ELISA using a generic biotin-labelled probe for detection of the presence of mucosal HPV sequences in the sample [30]. Samples positive for HPV are then genotyped using the LINEAR ARRAY® HPV Genotyping Test (Roche) for the simultaneous detection of up to 37 HPV genotypes (6, 11, 16, 18, 26, 31, 33, 35, 39, 40, 42, 45, 51, 52, 53, 54, 55, 56, 58, 59, 61, 62, 64, 66, 67, 68, 69, 70, 71, 72, 73, 81, 82, 83, 84, IS39, and CP6108) with the modification of using a BeeBlot (Bee Robotics Ltd, Gwynedd, United Kingdom) machine-validated by our laboratory [31-33]. HPV genotyping profiles are manually interpreted and verified using the HPV reference guide provided with each test kit. Any sample positive for the HPV 52/33/35/58 probe line on LA are further tested to confirm the presence or absence of HPV52 [34].

Participants identified as positive for HPV-16 or HPV-18 are contacted and advised by a senior researcher regarding appropriate follow-up according to an ethics approved protocol used in a previous Australia-wide HPV genotyping study [21], with consideration of their Pap screening history, vaccination status, and any other relevant information. If they have had a recent normal Pap they are offered a repeat HPV DNA and Pap in 12 months’ time. If they have had a recent abnormal Pap they are advised to see their clinician for further follow-up, or referred appropriately in line with current clinical guidelines.

#### Sample size and power

A number of factors were considered in the calculation of the sample size required for the population cohort study, including age at vaccination, vaccination coverage, HPV-16/18 prevalence in the non-vaccinated population, and vaccine efficacy against persistent HPV infection, which were used to estimate the likely vaccine effectiveness that would be observed. In regards to age, there were two populations to consider, being the school-vaccinated population (women aged 12–17 in 2007, 16–21 in 2011) and the GP/community-vaccinated women (aged 18–22 in 2007, 22–26 in 2011). In the school-vaccinated population we expect ~80% coverage (at least one dose [15]); whilst in the GP-vaccinated population we expect ~65% coverage (at least one dose). Results from the Women’s HPV Indigenous Non-Indigenous Urban Rural Study (WHINURS), an Australian study of cervical HPV prevalence in women presenting for a Pap test prior to the vaccination program, indicated HPV-16/18 prevalence of 27.5% for women aged 16–21 and 15.5% for women aged 22–26, which were taken as indicative prevalence for the current sexually active non-vaccinated population. Vaccine trial data using the end point of persistent HPV16/18 at 6 months showed a vaccine efficacy of 78% [35]. With these factors taken into consideration, a total sample size of 1569 (890 women aged 16–21 and 679 women aged 22–26), is required to estimate the anticipated post-vaccination HPV16 prevalence of 10.3% in women 16–21 and 7.6% in women 22–26, with an absolute precision of +/−2 % each, with alpha set at 0.05.

### Methods: sub study B

#### Biopsies

All cervical biopsies reported either at the Royal Women’s Hospital (RWH) Department of Pathology or Victorian Cytology Service (VCS) Pathology are being screened for eligibility for study. Biopsies are considered eligible for inclusion if CIN3 or ACIS is identified in the biopsy and subjects were born after 30^th^ June 1981.

#### HPV vaccination record

Participant’s HPV vaccination histories are obtained from the NHVPR, where participants have provided consent to access their records as part of their clinical care. Self-reported vaccine status including number of doses and time of vaccination are requested for RWH participants at the time of their dysplasia assessment.

#### Serial sections

Serial sections are taken from each case, 5 sections per block. Cross-contamination is minimised by ensuring that the microtome stage and forceps were cleaned with Para Kleaner (United Biosciences, Carindale, Qld, Australia) followed by ethanol between blocks, a fresh blade, and a new water container for floating of sections are used for each case. For each biopsy, the first and last sections are cut at 3μM thickness onto standard glass slides, H&E stained and cover-slipped. The first intervening section is cut at 8μM and placed onto a PEN membrane slide (Life Technologies, Grand Island, NY). This slide remains unstained and not cover-slipped until ready for Laser Capture Microdissection (LCM). The stained H&E slides are scanned using an Aperio ScanScope (Aperio, Vista, CA) slide scanner at 10x resolution, and reviewed by a pathologist who annotates CIN3 regions to be excised by LCM, using the Aperio ImageScope software (Aperio).

The second and third intervening sections are cut at 8μM and placed inside a sterile 1.5ml Eppendorf tube for DNA extraction and subsequent HPV detection. These sections are firstly de-waxed by adding 800 μl Histolene reagent and 400μl 100% ethanol directly to the sterile 1.5ml Eppendorf tube. The supernatant is removed, the section washed in 100% ethanol and dried briefly at room temperature before digestion in Tissue Lysis Buffer (Roche) and Proteinase K and incubated at 55°C for 1 h and overnight at 37°C. The entire reaction is then loaded onto the MagNA Pure instrument (Roche) for automated DNA extraction. Subsequent HPV detection is performed using the RHA kit HPV SPF10-LiPA25, version 1 (Labo Bio-medical Products, Rijswijk, Netherlands).

### LCM

Each unstained PEN Membrane slide is individually and manually de-waxed to minimise cross-contamination of tissue. The slide is de-waxed in two washes of 100% xylene (5 min each) and stored in 100% ethanol for LCM analysis. This is performed on the day of LCM analysis to minimise over-fixing the tissue to the slides. LCM is performed using an Arcturus Veritas Microdissection Instrument (Life Technologies,), using CapSure Macro LCM Caps (Life Technologies).

DNA extraction from LCM captured tissue is performed using the PicoPure DNA Extraction Kit (Life Technologies). Briefly, 50μl of PicoPure Reagent is added to each CapSure Macro LCM Cap and incubated at 65°C for 13 hours, followed by 95°C for 15 min to inactivate the Reagent. Following incubation, HPV detection is conducted using the RHA kit HPV SPF10-LiPA25, version 1 (Labo Bio-medical Products).

#### Sample size & power

Baseline data for this study comes from a pre-vaccine era study of women treated for CIN3, with HPV typing performed by our laboratory on a concurrently collected cervical smear. The prevalence of HPV-16 or 18 for 78 women aged ≤24 was 66.7% and 9.0% respectively, and 69.2% overall. For the 102 women aged 25–29 it was 60.8% or 6.9% respectively and 63.7% overall. For women aged <25 years, a sample 217 of women will have 80% power to detect a fall from 67% to 50%. For women aged 25–29 years a sample of 276 women will have 80% power to detect a fall from 61% to 46%. Therefore, the total sample size was calculated to be 500 (220 women aged <25 and 280 women aged 25–30). If funding allows we will retrospectively analyse a separate random sample of pre-vaccine CIN3 specimens from young women using the current LCM methodology to provide an even more robust comparative baseline.

## Discussion

Australia is a world leader in the early implementation of HPV vaccine programs, with approximately 83% of eligible females in the target population of 12–13 year olds having had at least one dose of the vaccine, and 73% having completed the 3 dose program [15]. With the successful uptake of the vaccine, in combination with robust national registries and databases (for cytology, cancer and HPV vaccine), Australia is well placed to gain a clear and early insight into the effectiveness of the HPV vaccine in reducing the prevalence of HPV in young women, and any subsequent reduction in the prevalence of pre-cancerous cervical lesions, specifically CIN3. Beyond the translational benefits to Victoria and Australia, the demonstration of successful ‘real world’ outcomes of a publically funded population based program will be valuable to other countries, especially countries who are not in the position to conduct their own studies, due to low HPV vaccination uptake or the absence of vaccine registries.

Early understanding of the prevalence of HPV genotypes after vaccine implementation in the real world, rather than in the restricted population of phase 3 vaccine trials, will help refine estimates of the eventual impact and cost-effectiveness of universal HPV vaccination as a strategy to prevent cervical cancer. This data will be important for refining models of HPV infection and cervical disease to inform the renewal of Australia’s national cervical screening program [36].

In addition, this study will provide key insights into the motivation for cervical screening behaviours in young women post-vaccination. A lack of awareness of young women that 30% of cervical cancers and 50% of high grade abnormalities will not be prevented by the vaccine could result in a decline in participation in cervical screening, and some loss of trust in the HPV vaccination program within the general community when cervical abnormalities are diagnosed among vaccinated women [37]. For the last decade there has been a gradual decline in participation in the National Cervical Screening Program among young women [38]. There is a concern that this decline may be exacerbated by a misperception by young women that the vaccine prevents all cervical cancer, and the population cohort study will identify if this is indeed the case.

### Potential for vaccine cross-protection for related HPV types

Whilst natural induced immunity is type-specific, it is noteworthy that vaccine-induced cross-protection against non-vaccine HPV types that are phylogenetically-related, has been seen in recent phase III trials. The PATRICIA trial showed cross-protective efficacy against HPV-33, HPV-31, HPV-45 and HPV-51 following vaccination with Cervarix® [39]. Another recent study compared HPV-31 and HPV-45 seropositivity rates following vaccination with either Cervarix® or Gardasil®, and demonstrated that induced seropositivity of both these types was sustained for 24 months following vaccination with either treatment [40]. Given that 80% of cervical cancer cases worldwide can be attributed to the four HPV types 16, 18, 31 and 45 [41,42]; this cross-protection may offer an important additional effect, although the durability of any such cross protection is unknown. It will be of interest to assess whether there is a measurable decrease in a real-life setting in these related but non-targeted HPV types among vaccinated cohorts of women.

### Potential for a replacement of vaccine related types in cervical disease

An unresolved issue in relation to widespread HPV vaccination is whether a potential exists for the replacement of HPV-16 and 18 as the most prevalent oncogenic HPV types by other currently less common, but also strongly oncogenic, types. Current genetic and evolutionary understanding of HPV (i.e. its relative ecological stability over time) suggests such a development is unlikely, with the weight of evidence suggesting only a slight interaction, at most, between infecting HPV types [43-45]. However there will be a need to monitor this issue closely due to the theoretical concerns that type-specific HPV vaccination may lead to an inadvertent increase in cervical disease caused by non-vaccine types through the removal of the ‘ecological niche’ occupied by types 16 and 18, resulting in competitive adaptation of other HPV types.

In summary, the VACCINE study will add significant data to the information already emerging from population based programs regarding the impact of HPV vaccination in early adopter populations.

## Abbreviations

ACIS: Adenocarcinoma in situ; CIN: Cervical Intraepithelial Neoplasia; HPV: Human Papillomavirus; LCM: Laser Capture Microdissection; NHVPR: National HPV Vaccination Program Register; PBS: Phosphate buffered saline; RWH: Royal Women’s Hospital; VACCINE: Vaccine Against Cervical Cancer Impact and Effectiveness; VAIN: Vaginal Intraepithelial Neoplasia; VCS: Victorian Cytology Service; VIN: Vulvar Intraepithelial Neoplasia; WHINURS: Women’s HPV Indigenous Non-Indigenous Urban Rural Study; WHO: World Health Organization.

## Competing interests

JMLB, DMG, MS, and SMG were partner investigators on an Australian Research Council Linkage Grant 2008–2011 on which CSL Biotherapies was a partner organisation. SMG has received funding through her institution to conduct HPV vaccine studies for MSD and GSK, advisory board and lecture fees and grant support from CSL and GlaxoSmithKline, and Sanofi Pasteur and is a member of the Merck Global Advisory Board plus the Merck Scientific Advisory Committee for HPV.

## Authors’ contributions

EJY is the project manager and data manager for the study, and is the primary author of this manuscript. SMG, chief investigator of the study, conceived the study and was involved in study design, study coordination and helped to draft this manuscript. SNT has overseen all laboratory aspects of the study, including study design and data collection, and drafted the methodology section of this manuscript. JMLB was involved in study design, data analysis and helped to draft this manuscript. MP, DG, JDW, YJ were involved in study design. MS, JP were involved in study design, planning and co-ordination of histology processing and pathologic review of all study cases for sub study B. JT was involved in the study design for sub study B. CDW is involved in the coordination of sub study B. All authors read and approved this manuscript.

## Pre-publication history

The pre-publication history for this paper can be accessed here:

http://www.biomedcentral.com/1471-2407/13/296/prepub
